# Editorial for the Special Issue on Microdevices and Microsystems for Cell Manipulation

**DOI:** 10.3390/mi8090276

**Published:** 2017-09-12

**Authors:** Wenqi Hu, Aaron T. Ohta

**Affiliations:** 1 Max Planck Institute for Intelligent Systems, 70569 Stuttgart, Germany; wenqi@is.mpg.de; 2 Department of Electrical Engineering, University of Hawaii at Manoa, Honolulu, HI 96822, USA

Microfabricated devices and systems capable of micromanipulation are well-suited for the manipulation of cells. These technologies are capable of a variety of functions, including cell trapping, cell sorting, cell surgery, and cell culturing, often at single-cell or sub-cellular resolution. The functionalities enabled by these microdevices and microsystems are relevant to many areas of biomedical research, including tissue engineering, cellular therapeutics, drug discovery, and diagnostics. This special issue of *Micromachines*, entitled “Microdevices and Microsystems for Cell Manipulation”, contains 11 papers (seven articles, three reviews, and one letter) highlighting recent advances in the field of cellular manipulation. 

In the areas of cell trapping, cell sorting, and cell characterization, Yousuff et al. reviews many types of microfluidic systems that are capable of sorting cells based on various characteristics [1]. This review includes cell sorters that use labels or surface markers, as well as the cell’s size, shape, deformability, density, compressibility, electrical properties, and magnetic properties to distinguish between cells within a sample. Work by Wang et al. is also aimed at characterizing cells; an atomic force microscope was used to mechanically deform cells, and the authors use this information to develop a dynamic model of the cell’s viscoelastic properties [2]. This information can also be used to distinguish between various cell types. The trapping and transportation of single cells using optically controlled microbubbles was demonstrated by Fan et al. [3]. This system uses an optically generated thermocapillary force to trap the cells. Dai et al. also used opto-thermally generated bubbles, but vibrated the bubbles to enhance the trapping force [4]. The trapping and transportation of multiple micro-objects using a single bubble was demonstrated, as well as the transportation of mammalian cells and small multicellular algae. 

This issue has five papers in the field of cell surgery. Chan et al. used electrical current as feedback to improve the automated microinjection of cells [5]. The effectiveness of the method on neuronal cells was verified by monitoring the injection process and studying the ion channel activities. Another two works on automated cell surgery are from Xie et al. Their first article discusses a visual-servo microrobotic system with both cell autofocusing and an adaptive visual processing algorithm [6], and achieved a 100% microinjection success rate on zebrafish embryos. Their second letter focuses on the challenge of cell fixation during automated cell injection [7]. They used a PDMS microarray cylinder to control the contact force between cells and the material, achieving a microinjection success rate over 80%. Cell lysis is also a crucial process to extract useful information from the cell interior. In the work of Fan et al. [3], laser-induced microbubbles and their associated microstreams were used to rupture targeted cell membranes and perform single-cell lysis. Other various cell lysis techniques, from macro- to microscale, are reviewed by Islam et al. [8]. This review paper describes the advantages and disadvantages of each technique and compares cell lysis methods applicable to microfluidic platforms. 

This issue also contains two papers in the field of cell culturing. Graphene oxide (GO) is suitable for cell growth due to its feature of high hydrophilicity and protein absorption. Kim et al. used meniscus-dragging deposition to fabricate GO micropatterns that affect the distance, speed, and directionality of cell migration [9]. Cell culturing in three-dimensional (3D) scaffolds is expected to significantly impact the fields of drug-screening and tissue engineering. In the work of Liu et al. electrodeposition was used to synthesize alginate hydrogel microstructures in arbitrary shapes, and the assembly of 3D hydrogel blocks was achieved [10]. This platform offers a way for researchers to synthesize complex 3D hydrogel structures for use in tissue engineering. Finally, in the review paper by Vadivelu et al. the use of cell spheroids to culture 3D tissue is surveyed and discussed, along with directions for future development in this area [11].

We would like to thank all the authors for their submissions to this special issue. We also thank all the reviewers for dedicating their time and helping to ensure the quality of the submitted papers.
